# Supplementary material for: Dynamic nanoscale architecture of synaptic vesicle fusion in mouse hippocampal neurons
Source: Nat Commun. 2025 Dec 13;16:11131. doi: 10.1038/s41467-025-67291-6 (PMC12706093; doi:10.1038/s41467-025-67291-6)
Supplement: Supplementary file 1 — Supplementary Information [file 41467_2025_67291_MOESM1_ESM.pdf]

Dynamic nanoscale architecture of synaptic vesicle fusion in mouse hippocampal neurons

Jana Kroll[1,2,3,#], Uljana Kravčenko[4,5], Mohsen Sadeghi[6,7], Christoph A. Diebolder[8,9], Lia Ivanov[1], Małgorzata Lubas[1], Thiemo Sprink[8,9], Magdalena Schacherl[10], Mikhail Kudryashev[4,10], Christian Rosenmund[1,#]

Supplementary Figures

- Suppl. Fig. 1: Assessment of action potential induction efficacies of three Channelrhodopsin-2 variants
- Suppl. Fig. 2: Banker culture of mouse hippocampal neurons on EM grids
- Suppl. Fig. 3: Setup and timing of optogenetic plunge freezer
- Suppl. Fig. 4: Live-imaging of fluorescent activity sensors
- Suppl. Fig. 5: Correlation of iGluSnFR3 cryo-confocal microscopy and EM of optogenetically stimulated neurons
- Suppl. Fig. 6: Examples of putative fusion events that were excluded
- Suppl. Fig. 7: Criteria for the classification of membrane rearrangements
- Suppl. Fig. 8: Ultrastructural characteristics of putative SV fusion intermediates
- Suppl. Fig. 9: Markov state models of SV fusion progression
- Suppl. Fig. 10: Coarse-grained simulation of SV approximation
- Suppl. Fig. 11: Distributions and tethering of SVs in stimulated and control synapses
- Suppl. Fig. 12: Additional examples of tethered SVs

Supplementary Tables

- Suppl. Table 1: Viral constructs

Supplementary methods

- Electrophysiological recordings
- Immunocytochemistry
- synGCamp6f and iGluSnFR live imaging
- Live imaging analysis
- Calculation of theoretical release probability based on SV distribution
- Virus constructs
- Markov state model
- Subtomogram averaging of fusion states

MD Simulation Checklist

Supplementary references

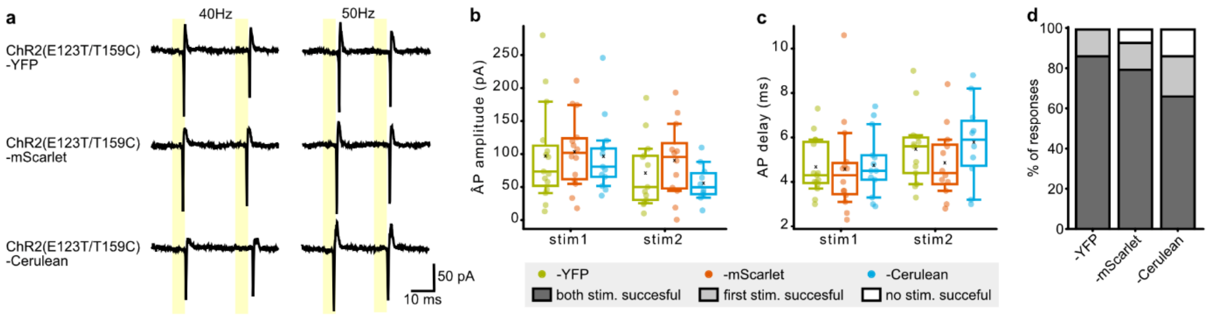

Suppl. Fig. 1: Assessment of action potential induction efficacies of three Channelrhodopsin-2 variants. (**a**) Exemplary light-induced responses to 40 Hz and 50 Hz paired pulses of ChR2 variants measured in cell attached patch clamp recording mode, light pulse duration 5 ms. (**b**) Action potential amplitudes in response to first and second light pulse at 40 Hz stimulation. (**c**) Delay between light onset and action potential induction for first and second light pulse at 40 Hz stimulation. (**d**) Success rate at 40 Hz stimulation. Dark gray: both stimuli successful, light gray: only first stimulus successful, white: stimulation not successful. ChR2(E123T/T159C)-YFP N = 15 cells, ChR2(E123T/T159C)-mScarlet N = 14 cells, ChR2(E123T/T159C)-Cerulean N = 13 cells. Box plots indicate median, 25% and 75% quartiles, whiskers indicate 10-90% percentiles. The mean is marked with an x. Source data are provided as a Source Data file.

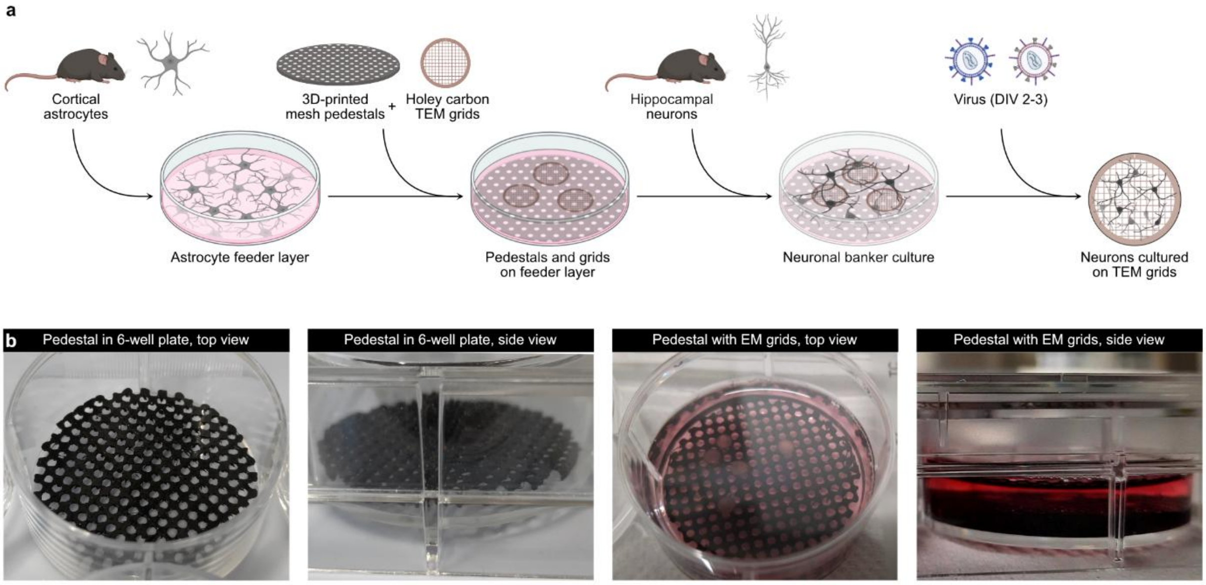

Suppl. Fig. 2: Banker culture of mouse hippocampal neurons on EM grids. (**a**) Schematic illustration showing the individual steps to culture neurons on EM grids. A feeder layer of mouse cortical astrocytes is seeded on 6-well plates and cultured for one week. 3D-printed mesh pedestals and glow-discharged, coated holey carbon grids are placed on top of the astrocyte feeder layer. Mouse hippocampal neurons are seeded and cultured for additional two weeks, viral constructs are added on neuronal day in vitro (DIV) 2-3. (**b**) Images of 3D-printed mesh pedestals in a 6-well plate without (left) or with (right) cell culture medium and EM grids. Panel a was created in BioRender. Kudryashev, M. (2025) https://BioRender.com/bx46pp6.

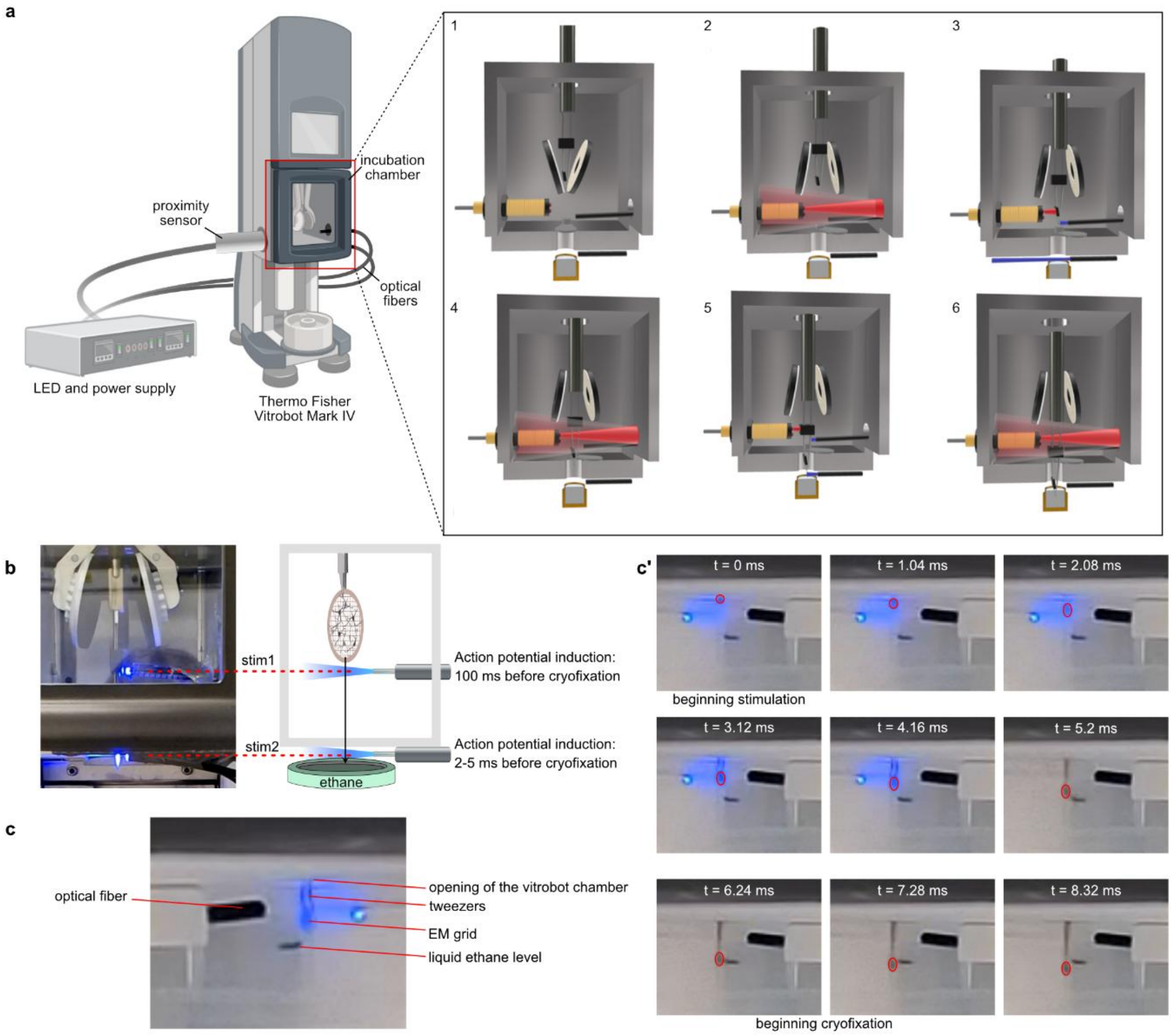

Suppl. Fig. 3: Setup and timing of optogenetic plunge freezer. (**a**) Schematic illustration of a Vitrobot Mark IV plunge freezer equipped with an LED and two optical fibers reaching inside and below the incubation chamber. The on-time of the LED is controlled with a proximity sensor. During grid loading and blotting, the LED is switched off (panel 1). As soon as the proximity sensor is activated by markers attached to the tweezers, the LED is switched on and blue light illuminates the path of the plunging EM grid (panel 2). The first light pulse is applied inside the chamber (panel 3), the second light pulse is applied below the chamber (panel 5). Between the two pulses (panel 4) and after the second pulse (panel 6), the LED is switched off. (**b**) Image of the Vitrobot chamber with two optical fibers when the LED is switched on. The timing between first light pulse and cryofixation in liquid ethane can be varied depending on the position of the optical fiber. For our experiments, we selected a time of ~ 100 ms between light pulse and freezing. (**c** and **c'**) Images of high-speed camera (frame rate 960 fps) capturing the illumination of an EM grid and the subsequent cryofixation, which is indicated by a black horizontal line at the level of liquid ethane (c). The illumination for optogenetic stimulation starts 6-8 ms before cryofixation. Taking into account that the delay between light onset and action potential induction was 3.9 - 5.9 ms for ChR2(E123T/T159C)-YFP, most action potentials were induced approximately 2-5 ms before cryofixation (c'). Panels a and b were created in BioRender. Kudryashev, M. (2025) https://BioRender.com/x071e11 and Kudryashev, M. (2025) https://BioRender.com/j5cdwwd.

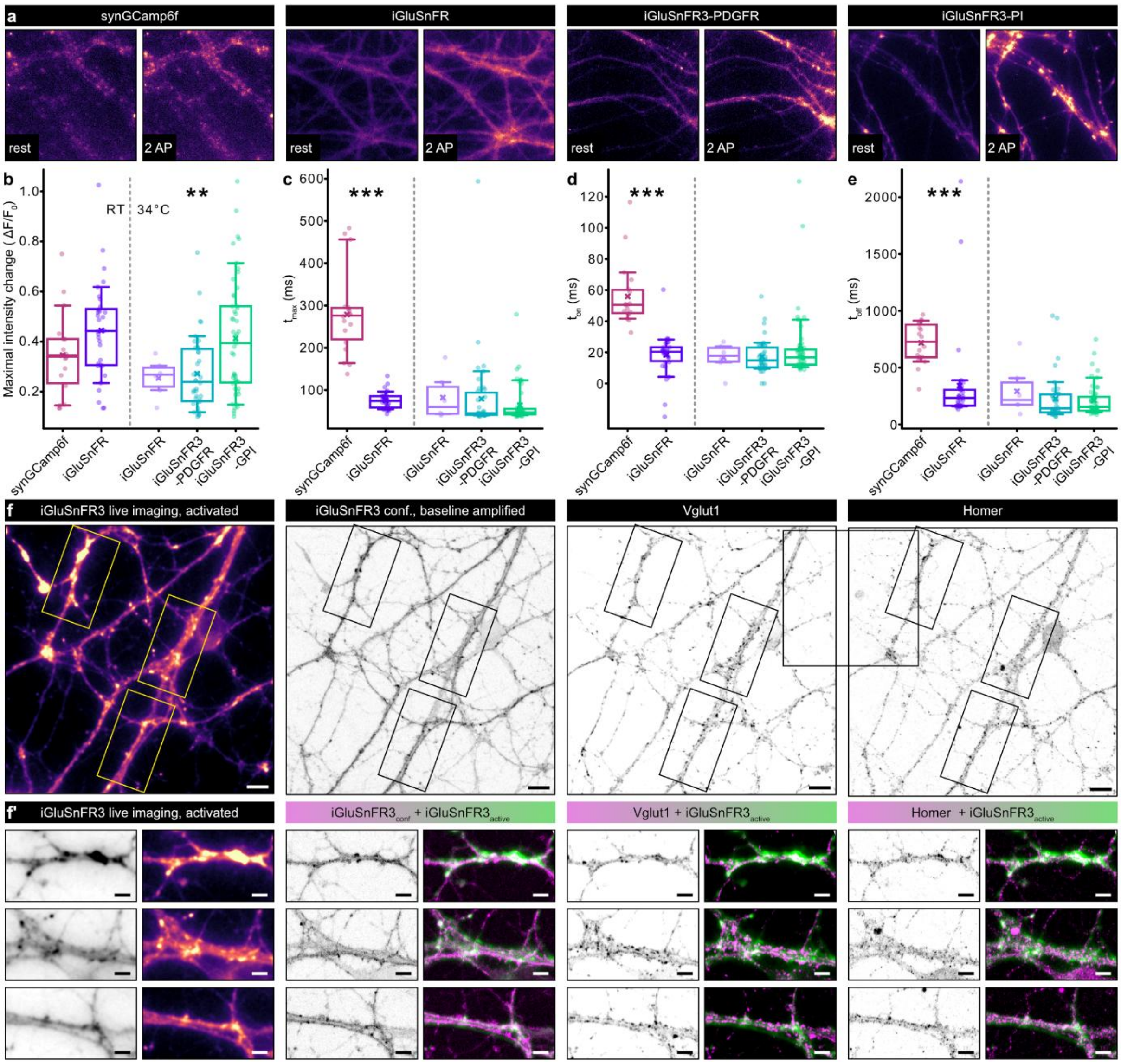

Suppl. Fig. 4: Live-imaging of fluorescent activity sensors. (**a**) Exemplary micrographs showing the activity sensors synGcamp6f, iGluSnFR, iGluSnFR3-PDGFR, and iGluSnFR3-GPI at rest and after application of one (synGcamp6f) action potential (AP) or two APs with a frequency of 40 Hz. (**b**) Maximal fluorescence intensity changes of the four activity sensors. (**c**) Delay between first action potential and time point with maximal fluorescence intensity. (**d**) Rise time until 50% of the maximal fluorescence intensity are reached. (**e**) Decay time until the fluorescence intensity decreased to 50% of the maximal intensity. (b-e) synGCamp6f and iGluSnFR (left boxplot) were recorded at room temperature, iGluSnFR (right boxplot) and both iGluSnFR3 constructs were recorded at a near-physiological temperature of 34°C. Box plots indicate median, 25% and 75% quartiles, whiskers reach from 10-90% of data points. The x represents the mean. ** $p < 0.01$, *** $p < 0.001$. synGCamp6f N = 18 ROIs/ 3 cultures, iGluSnFR (RT) N = 32 ROIs/ 2 cultures, iGluSnFR (34°C) N = 6 ROIs/ 3 cultures, iGluSnFR3-PDGFR N = 38 ROIs/ 5 cultures, iGluSnFR3-GPI N = 51 ROIs/ 5 cultures. (**f**) Correlation of iGluSnFR3-GPI live imaging and *post hoc* immunostaining using antibodies against Vglut1 and Homer. Scale bars 10 µm. (**f'**) Zoom-ins to individual neurites and overlay of the iGluSnFR3 live imaging signal with iGluSnFR3 baseline (left), Vglut1 (middle) and Homer (right). Scale bars 5 µm. Source data are provided as a Source Data file.

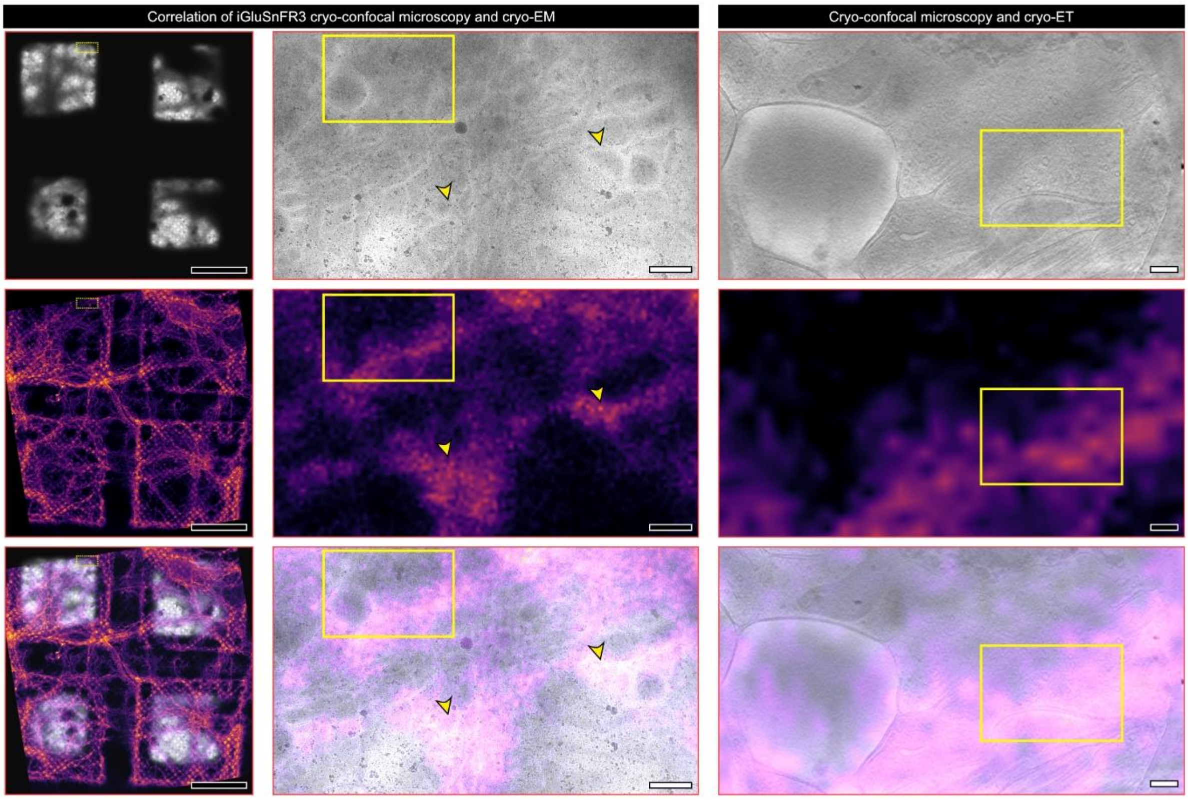

Suppl. Fig. 5: Correlation of iGluSnFR3 cryo-confocal microscopy and EM of optogenetically stimulated neurons. The top row contains TEM images (left, middle column) or tomogram slices (right column), yellow arrowheads and boxes point to regions with a high abundance of putative synaptic boutons. The middle row contains maximum intensity z-projections of cryo-confocal micrographs of the glutamate sensor iGluSnFR3. Here, the arrowheads and boxes point to areas with comparatively high fluorescence intensity, which indicates glutamate release. The bottom row contains overlays of fluorescence and EM signals. Scale bars left: 50 µm, middle: 500 nm, right: 200 nm.

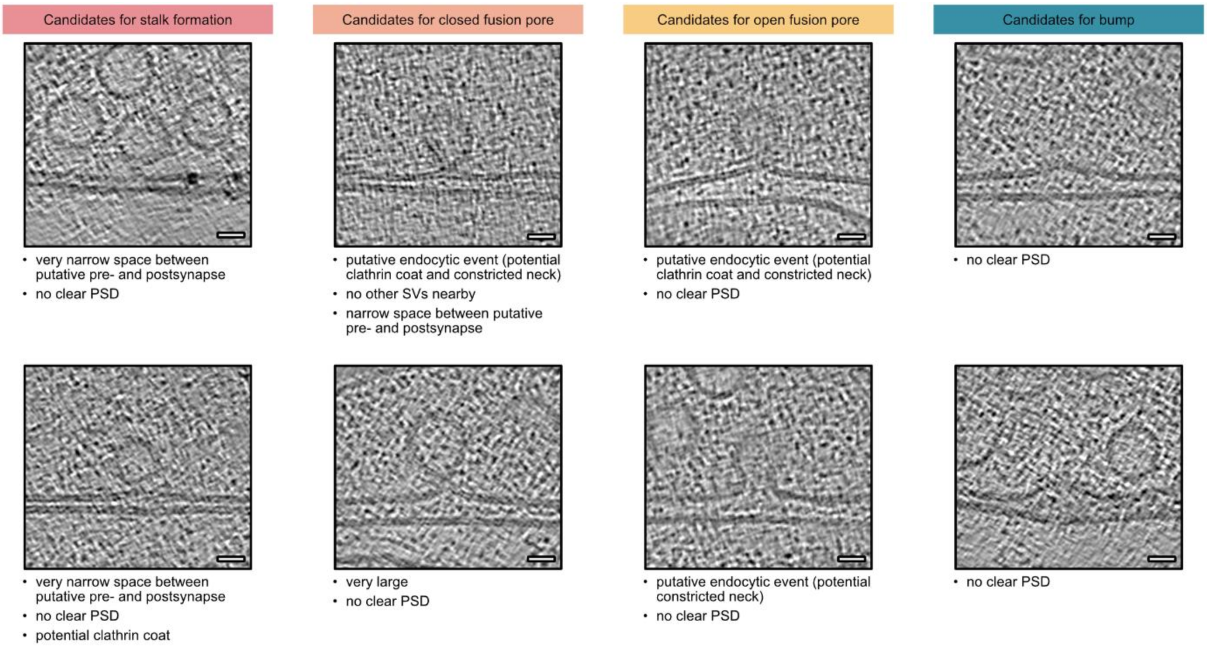

Suppl. Fig. 6: Examples of putative fusion events that were excluded. Tomogram slices of candidates for stalk formation, closed and open fusion pores and bumps. These and similar putative fusion events were excluded from the analysis because no postsynaptic density was visible, because the synaptic cleft was very narrow, or because a halo resembling a clathrin coat was visible. Scale bars 20 nm.

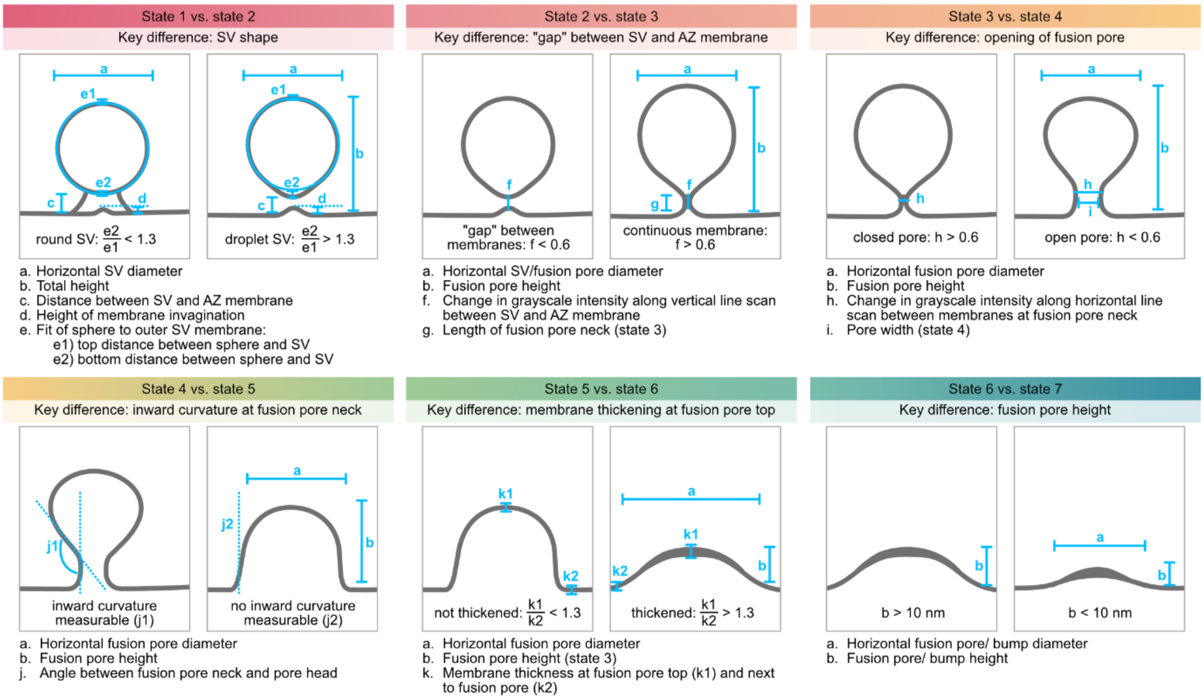

Suppl. Fig. 7: Criteria for the classification of membrane rearrangements. Schematic illustrations of morphometric measurements, which were performed to classify membrane rearrangements referring to fusion states (state 2-6), a putative pre-fusion state (state 1) and a putative post-fusion state (state 7). Key differences to distinguish between states are highlighted. Details about the individual measurements are explained in the Methods section. Source data are provided as a Source Data file.

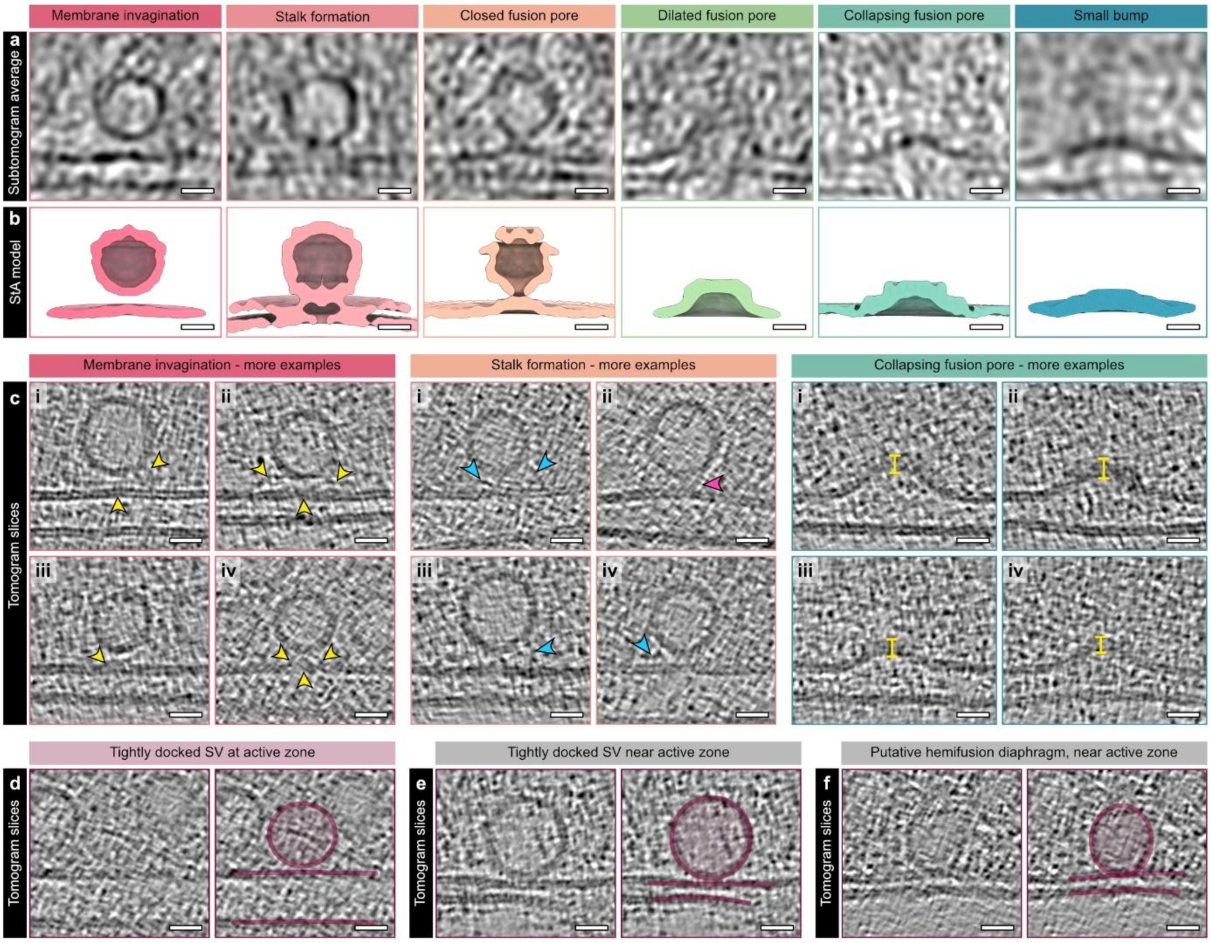

Suppl. Fig. 8: Ultrastructural characteristics of putative SV fusion intermediates. (**a**) Subtomogram averaging of putative membrane rearrangements during SV fusion. Membrane invagination (state 1) N = 5 averaged subtomograms, stalk formation (state 2) N = 8, closed fusion pore (state 3) N = 4, dilating fusion pore (state 5) N = 5, collapsing fusion pore (state 6) N = 6, small bump (state 7) N = 10 averaged subtomograms. (**b**) C61-symmetrized isosurfaces of the subtomogram averages of panel a. (**c**) Additional examples of SVs with membrane invaginations, stalk formations, and collapsing fusion pores. Not only below tethered SVs but also during stalk formation, only slight invaginations of the active zone membrane are visible (yellow arrowheads). During stalk formation, the space between SV and AZ membrane appeared partially blurry (pink arrowhead in example ii). Filaments were not only observed as tethers between the SV and the AZ membrane, but also attached to fusion events and reaching into the cytosol, as indicated by blue arrowheads. The top membrane of collapsing fusion pores was thicker than the AZ membrane (yellow lines). (**d**) Within all tomograms, only one example of a tightly docked SV at an AZ membrane was observed. (**e**) Additionally, a second tightly docked SV and a putative hemifusion diaphragm (**f**) were observed at membranes that were not opposed to a postsynaptic density, meaning not at an active zone. All scale bars 20 nm.

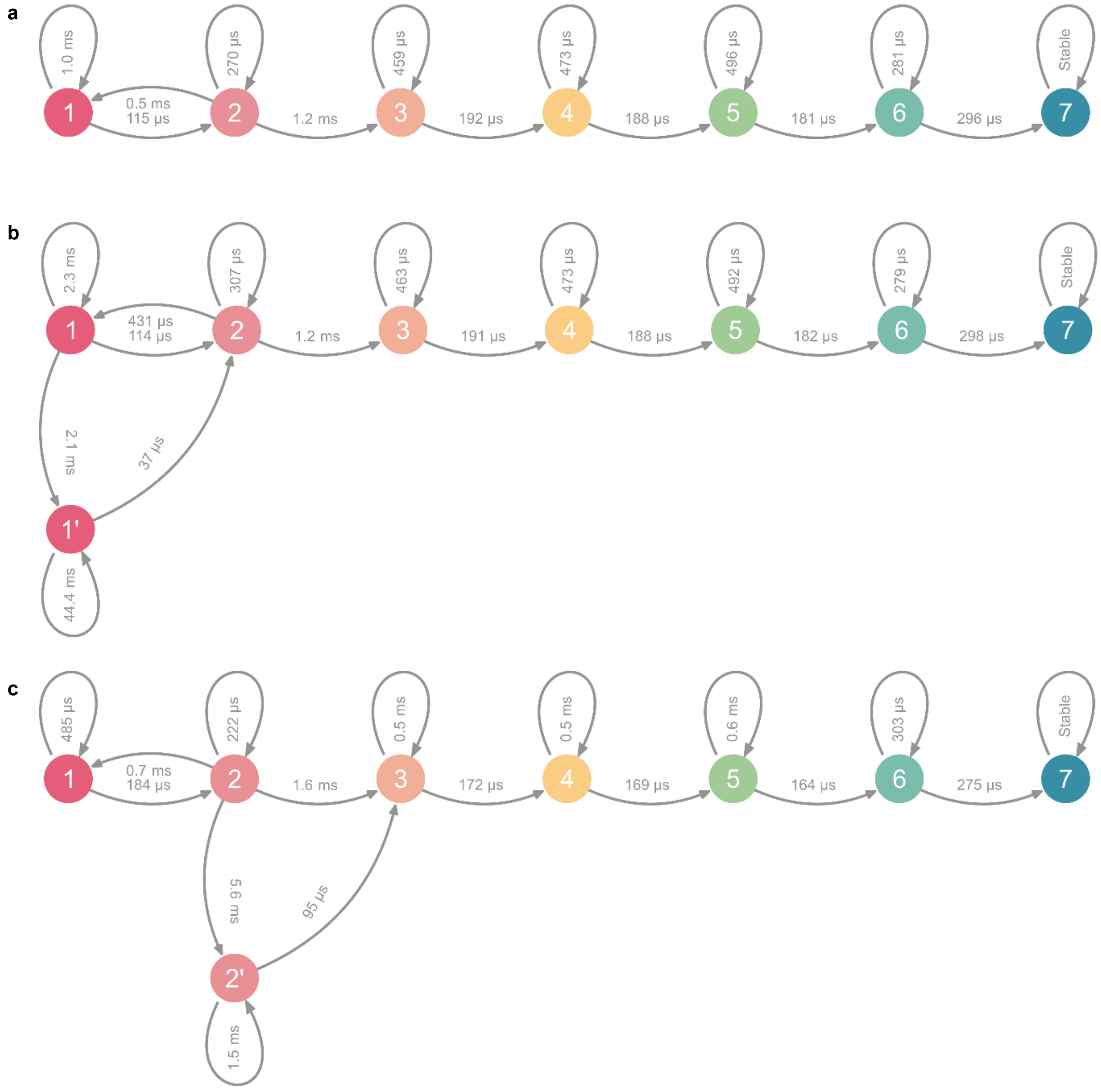

Suppl. Fig. 9: Markov state models of SV fusion progression. Kinetics of transitions between discrete states, as predicted by the Markov state models fitted to the observed state statistics in imaged samples. Numbers and colors assigned to each state follow the scheme of Figs. 3a and 3f with the same sequence. Time values shown for each transition, as well as self-transitions, correspond to mean waiting times, with larger values highlighting less probable transitions. The graphs are shown for three Markov models in which, (**a**) a linear chain of forward transitions between states 1 to 7 is assumed, with only one reversible transitions possible between states 1 and 2, (**b**) similar to (a), with the auxiliary state 1' corresponding to the tight-docking configuration occurring between states 1 and 2, and (**c**) similar to (b), with the tight-docking state 2' occurring between states 2 and 3.

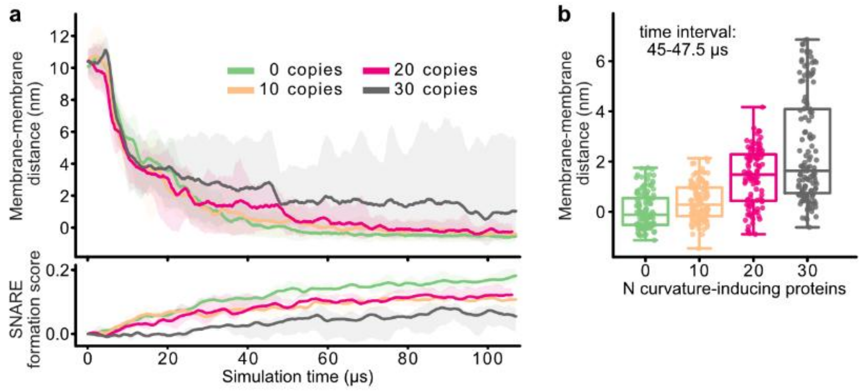

Suppl. Fig. 10: Coarse-grained simulation of SV approximation. (**a**) Top: vertical distance between the SV and AZ membranes, measured throughout the simulation, for simulations with the given copy numbers of curvature-inducing proteins in the AZ. Distance values are signed, with negative numbers pointing to partial penetration between particles forming the two membranes, as the fusion is not allowed. Error bands correspond to the full range of values measured in all simulation replicas. Bottom: time evolution of a score assigned to the successful formation of zippered SNARE complexes in each simulation (the legend is shared between top and bottom panels). The score is calculated based on the mean pairwise distance between v-SNARE and t-SNARE particles, with higher values pointing to more successful contacts. (**b)** Distribution of membrane-membrane distance measurements for simulations including different copy numbers of curvature-inducing proteins. Distances are sampled in all simulation replicas, during the given shared time interval. Box plots indicate median, 25% and 75% quartiles, whiskers span over all data points.

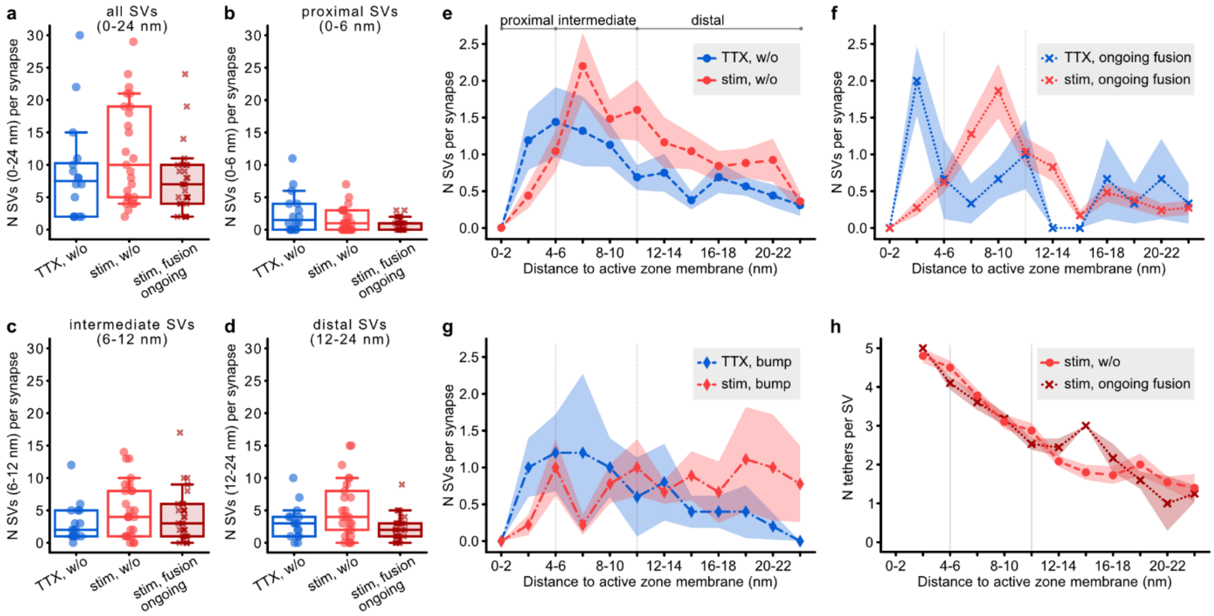

Suppl. Fig. 11: Distributions and tethering of SVs in stimulated and control synapses. (**a**) Number of SVs per synapse within a distance of 24 nm from the AZ membrane in TTX-treated synapses without membrane rearrangements (w/o), in stimulated synapses without membrane rearrangements, and in stimulated synapses with ongoing fusion. (**b**) Number of membrane-proximal SVs per synapse, maximal distance 6 nm. (**c**) Number of intermediate SVs per synapse, distance 6-12 nm. (**d**) Number of distal SVs per synapse, distance 12-24 nm. (**e**) Distribution of SVs in stimulated and TTX-treated synapses without membrane rearrangements. (**f**) Distribution of SVs in stimulated and TTX-treated synapses with ongoing fusion. (**g**) Distribution of SVs in stimulated and TTX-treated synapses with bumps. N TTX-treated synapses w/o = 16, N stimulated synapses w/o = 25, N stimulated synapses with ongoing fusion = 29 (for a-g). (**h**) Correlation of tether number and distance of the SV to the AZ membrane in stimulated synapses without membrane rearrangements (N = 145 SVs from 15 synapses) and in stimulated synapses with ongoing fusion (N = 114 SVs from 18 synapses). Box plots indicate median, 25% and 75% quartiles, whiskers indicate 10-90% percentiles. Data in line graphs are represented as mean ± SEM. Source data are provided as a Source Data file.

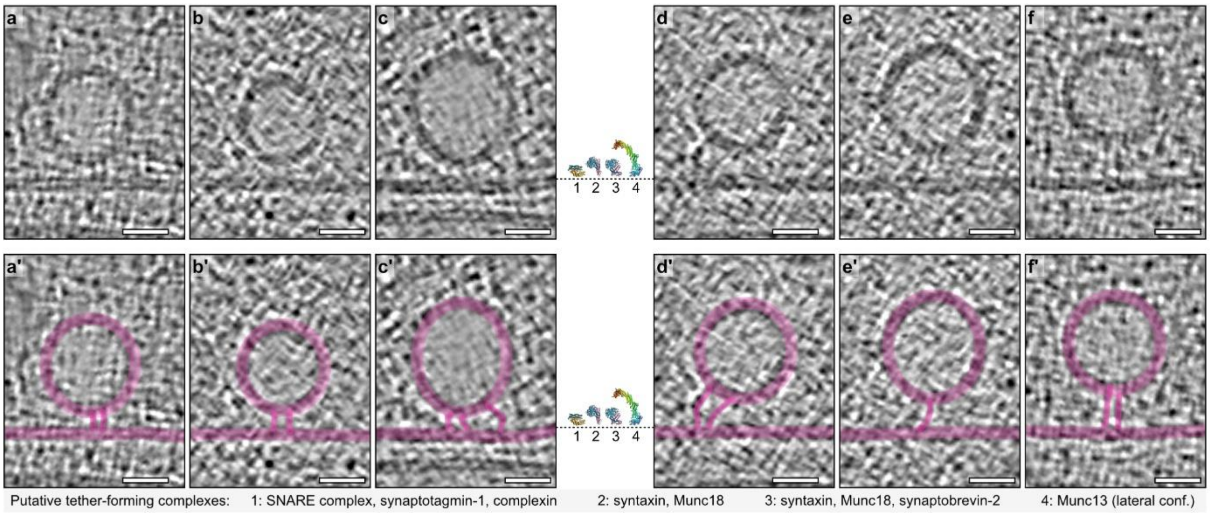

Suppl. Fig. 12: Additional examples of tethered SVs. Exemplary tomogram slices of membrane-proximal (a-c), intermediate (d-e), and distal SVs (f) with visible tethers linking to the AZ membrane. Tethers, SV and AZ membrane are highlighted in pink (a'-f'). Candidate proteins and complexes suggested to be involved in tether formation are depicted in scale: 1 assembled SNARE complex with synaptotagmin-1 and complexin (PDB: 5W5D[1]); 2 syntaxin and Munc18 (PDB: 4JEU[2]), 3 syntaxin, Munc18, and synaptobrevin-2 (PDB: 7UDB[3]); 4 Munc13 (lateral conformation, PDB: 7T7V[4]). Scale bars 20 nm.

Supplementary methods

Electrophysiological recordings

Cell-attached voltage-clamp recordings were performed on mass cultured neurons at room temperature at days in vitro 14-15. Currents were recorded using a Multiclamp 700B amplifier (Axon Instruments) controlled by Clampex 9 software (Molecular Devices). Membrane capacitance and series resistance were compensated by 70% und data filtered by low-pass Bessel filter at 3 kHz and sampled at 10 kHz using an Axon Digidata 1322A digitizer (Molecular Devices). A fast perfusion system (SF-77B; Warner Instruments) continuously perfused the neurons with the extracellular solution which contained the following (in mM): 140 NaCl, 2.4 KCl, 10 HEPES (Merck, NJ, USA), 10 glucose (Carl Roth, Karlsruhe, Germany), 2 CaCl2 (Sigma-Aldrich, St. Louis, USA), and 4 MgCl2 (Carl Roth) (~300 mOsm; pH 7.4). The extracellular solution was supplemented with NBQX (5 µM) and bicuculine (15 µM) to block network activity. Borosilicate glass pipette containing extracellular solution were kept at resistance 2–4 MOhm, and access resistance was compensated by 70%. Optical stimulation was provided by a 470nm LED coupled into the fluorescence port of the microscope (Olympus-IX71) and triggered for 5ms by a transistor-transistor logic (TTL) signal. To evoke AP trains, 40 and 50Hz light pulse trains were triggered every 5s.

Peak amplitude and delay of the responses were calculated using Axograph X (Axograph Scientific). Delay was measured from the light onset to the peak of the evoked action potential. For each cell, 3 trials of trains were averaged to determine the final values.

Immunocytochemistry

After live imaging, neurons were immediately fixed with 4% paraformaldehyde in phosphate-buffered saline (PBS) for 15 min followed by a brief wash in PBS. The cells were permeabilized using 0.1% triton x-100 in PBS (PBS-T), 3 x 10 min, and fluorescence was quenched using 100 mM glycine in PBS for 10 min. Unspecific binding sites were blocked with 5% normal donkey serum in PBS-T for 1h, primary antibodies diluted in blocking solution were incubated overnight. The cells were washed 3 x 10 min with PBS-T, secondary antibodies diluted in PBS-T were applied for 1h at room temperature. Again, the cells were washed 3 x 10 min with PBS-T and once with PBS for 5 min followed by mounting with mowiol. Primary antibodies: rabbit anti-Homer1 (Synaptic systems, #160 003, dilution 1:200) and guinea pig anti-Vglut1 (Synaptic systems, #135 304, dilution 1:4,000); secondary antibodies: Rhodamine Red donkey anti-rabbit IgG (Jackson ImmunoResearch, #711-295-152, dilution 1:1000) and Alexa Fluor 647 donkey anti-guinea pig IgG (Jackson ImmunoResearch, #706-605-148, dilution 1:1000).

Confocal stacks of regions, which were imaged during iGluSnFR3 live imaging beforehand, were acquired using a Leica SP5 confocal microscope with a 63x oil immersion objective, pixel size 0.12 µm and z-steps of 0.5 µm. Overlays of live imaging and maximum intensity z-projections of confocal stacks were generated manually using fiji.

synGCamp6f and iGluSnFR live imaging

Room temperature live imaging of neurons (DIV14-18) infected with synGCamp6f or iGluSnFR was performed on an inverted microscope (Olympus IX71) equipped with a 60x water immersion objective, Andor iXon camera (Oxford instruments), and 490 nm LED system (pE2; CoolLED). Cells were perfused with high-calcium extracellular solution containing the following: 140 mM NaCl, 2.4 mM KCl, 10 mM HEPES, 10 mM glucose, 4 mM CaCl2, and 1 mM MgCl2 (300 mOsm; pH 7.5). 6 µM NBQX and 30 µM bicuculline were added to block

neuronal network activity. Action potentials (2 ms depolarization) were induced using a field stimulation chamber (Warner Instruments), Multiclamp 700B amplifier, and an Axon Digidata 1550B digitizer controlled by Clampex 10 software (all Molecular Devices). To analyze the kinetics of the biosensors, single action potentials were induced and images were captured with an exposure time of 10 ms and a frame rate of 40 fps.

Live imaging analysis

Live imaging recordings were analyzed using GraphPad Prism (room temperature) and python (elevated temperature). Stimulation and image acquisition times were extracted using axograph, fluorescence intensities of each micrograph were measured using fiji. Datasets recorded at room temperature and at elevated temperature were analyzed separately. For the analysis of synGCamp6f and iGluSnFR at room temperature, F_0 was calculated as the mean fluorescence intensity of the first four and the last four images of each recording. t_{max} was defined as the time point at which the fluorescence intensity reached its maximum. For the calculation of t_{on}, a sigmoidal curve was fitted to the fluorescence intensity values between the time point of stimulation and t_{max} using GraphPad Prism. t_{on} was defined as the time point at which 50% of t_{max} were reached and calculated using the sigmoidal fit. Likewise, t_{off} was defined as the timepoint, at which the fluorescence intensity after stimulation decreased to 50% of its maximum, and calculated using an exponential fit. Recordings with a fluorescence intensity change ΔF/F0 of less than 0.1 were excluded from the analysis (see Source Data for numbers of excluded recordings for each fluorescent sensor).

For the analysis of iGluSnFR constructs at elevated temperature, fluorescence intensity values were corrected for bleaching by fitting an exponential decay curve to the baseline signals before and after action potential induction. Although two APs at 40 Hz were applied, only the time point of the first AP was used for calculations. Definitions of t_{max}, t_{on} and t_{off} were the same as for the analysis of room temperature recordings. Likewise, a sigmoidal fit was used for the rise time and an exponential fit for the decay time. Again, recordings with ΔF/F0 of less than 0.1 were excluded.

Calculation of theoretical release probability based on SV distribution

Assuming that (1) the distribution of membrane-near SVs in synapses short after fusion resembles synapses during SV fusion, and (2) that the distribution of SVs in non-releasing synapses resembles the control condition (TTX):

(1) $N(proxSVs)_{fusion} = N(proxSVs)_{postfusion} = 0.89$

(2) $N(proxSVs)_{non\text{-}releasing} = N(proxSVs)_{TTX} = 2.54$

And (3) that the group of stimulated synapses without membrane rearrangements contains fraction x of postfusion synapses and fraction y of non-releasing synapses:

(3) $x * N(proxSVs)_{postfusion} + y * N(proxSVs)_{non\text{-}releasing} = 1.48$; with $x + y = 1$

We can calculate the theoretical fractions of postfusion synapses x = 0.83 and non-releasing synapses y = 0.17 within the group of stimulated synapses without membrane rearrangements.

Taking into account that 46% of all stimulated synapses did not show membrane rearrangements, the overall theoretical fraction of non-releasing synapses is 0.08, the

theoretical release probability would thus be 0.92 based on the distribution of membrane-proximal SVs.

Virus constructs

Virus production (lentivirus and AAV) was performed by the Viral Core Facility of the Charité – Universitätsmedizin Berlin (vcf.charite.de).

Plasmid identifier	Name	Insert	Site-directed mutant	Tag	Source
Lentiviruses					
BL-347	f(syn)ChR2(E123T/T159C)-YFP-w	Channelrhodopsin-2	(E123T/T159C)	YFP	VCF, Addgene #35511[5,6]
BL-1847	f(syn)ChR2-E123T/T159C-mscarlet-w	Channelrhodopsin-2	(E123T/T159C)	mScarlet	This study
BL-1848	f(syn)ChR2-E123T/T159C-Cerulean3-w	Channelrhodopsin-2	(E123T/T159C)	Cerulean	This study
BL-700	f(syn)SynGCamp6f-w	SynGCamp6f	-	-	VCF, Addgene #40755[7,8]
Adeno-associated viruses					
AAV 61	syn iGLuSnFR wt WPRE3	iGLuSnFR	-	-	VCF, Addgene #41732[9]
AAV 336	pAAV.hSyn.iGluSnFR3.v857.PDGFR	iGluSnFR3	Version857	PDGFR	VCF, Addgene #178329[10]
AAV 337	pAAV.hSyn.iGluSnFR3.v857.GPI	iGluSnFR3	Version857	GPI	VCF, Addgene #178331[10]

Suppl. Table 1: Viral constructs.

Markov state model

A discrete-time Markov state model represents memoryless transitions between discrete states in a series of discrete timesteps. The memoryless property implies that the probability of landing in a new state in the next timestep only depends on the current state. Thus, if the vector $\mathbf{p}(t) = (p_1(t), \ldots, p_N(t))$ represents probabilities $p_1(t), \ldots, p_N(t)$ of finding the system in states $1, \ldots, N$ at time t, in the most general way, the dynamics of the Markov chain can be summarized as $\mathbf{p}(t + \Delta t) = \mathbf{p}(t)\mathbf{T}(\Delta t)$ where $\mathbf{T}(\Delta t)$ is the transition matrix. Each component $T_{ij}(\Delta t)$ of the transition matrix would thus correspond to the transition probability of going from state i to state j in one timestep Δt. With n sequential timesteps, the final probabilities are easily derived as $\mathbf{p}(t + n\Delta t) = \mathbf{p}(t)\mathbf{T}^n(\Delta t)$.

To construct Markov state models, we have selected discrete states according to the designation in Fig. 3a, which are screened from the cryo-EM images based on the criteria defined in Suppl. Fig. 7. Apart from the rare tight-docking configuration, seven separate states are recognized, and their statistics obtained from a total of 86 studied samples (Source Data). The probability of finding the system in each state at the end stage can thus be estimated based on the number of observations containing that state. We additionally assume that all the systems start at the same state, 1, and undergo transitions for the same total duration of 2 ms. We discretize this time interval using a timestep of 0.2 ms. In all cases, the final state is made an absorbing state, i.e. when a system reaches state 7, it will remain there indefinitely.

Fitting of the Markov model to the data is achieved by comparing the predicted probabilities, $\mathbf{p}_{\text{predicted}}$, and the observed probabilities, $\mathbf{p}_{\text{observed}}$, and optimizing for T_{ij} components that minimize the objective $\|\mathbf{p}_{\text{predicted}} - \mathbf{p}_{\text{observed}}\|$, where $\|.\|$ denotes the vector norm. The optimization is done using the L-BFGS-B algorithm[11], with bounds $0 \le T_{ij} \le 1$ enforced on transition probabilities. We have used the L-BFGS-B implementation from the SciPy python package[12]. To achieve better results, for each case, several instances of the optimization are spawned with random initial points, and the best outcomes are chosen.

If we use a full transition matrix, the fitting would become highly underdetermined, considering that we only have access to N values of probability at the final stage of the dynamical process. We thus make simplifying assumptions about the sequence of events, with only few transitions allowed, as depicted in Suppl. Fig. 9. These assumptions translate to a sparse transition matrix with few components left to be determined. Because of the fact that we used a realistic total time for the Markov chain, we can easily translate the transition probabilities into mean waiting times, τ_{ij}, using the relation $T_{ij} = 1 - \exp(-\Delta t/\tau_{ij})$.

Subtomogram averaging of fusion states

For subtomogram averaging of fusion states, 4-times binned subtomograms (without denoising or IsoNet[13] correction) were used. For each state, corresponding subtomograms were manually aligned in *dynamo_gallery* to produce an initial average. This was used as an initial reference for a global alignment of all subtomograms. The subtomogram average for each category was performed separately in Dynamo[14]. Due to the low subtomogram number per class (4-10), averages were c61-symmetrized for the final representation in ChimeraX.

A high-order (61-fold) cyclic symmetry was applied to produce enough sampling points for generating a smooth rotational average around the vesicle-membrane fusion site. This approach assumes that vesicle fusion is, in principle, radially symmetric around the fusion axis,

even if individual events are heterogeneous. To define this axis, a small number of representative fusion events was manually aligned in Dynamo, using orthogonal (X/Y) and top-down (Z) views to identify the centers of the SV and the fusion pore. The axis was approximated as the vector connecting these two features, rather than being assumed to align with the tomogram's Z-axis (the axis of view).

MD Simulation Checklist

Reliability and reproducibility checklist for molecular dynamics simulations<br>*All boxes must be marked YES by acceptance unless "Response not needed if No".	Yes	No	Response (Please state where this information can be found in the text)
**1. Convergence of simulations and analysis**			
1a. Is an evaluation presented in the text to show that the property being measured has equilibrated in the simulations (*e.g.* time-course analysis)?	☒	☐	Suppl. Fig. 8
1b. Then, is it described in the text how simulations are split into equilibration and production runs and how much data were analyzed from production runs?	☒	☐	Under the *Simulation* subsection of the *Material and Methods.*
1c. Are there at least 3 simulations per simulation condition with statistical analysis?	☒	☐	We invoked 5 replicas per simulated scenario.
1d. Is evidence provided in the text that the simulation results presented are independent of initial configuration?	☒	☐	Simulation replicas were run with randomized initial configurations and results were consistent across replicas.
**2. Connection to experiments**			
2a. Are calculations provided that can connect to experiments (*e.g.* loss or gain in function from mutagenesis, binding assays, NMR chemical shifts, J-couplings, SAXS curves, interaction distances or FRET distances, structure factors, diffusion coefficients, bulk modulus and other mechanical properties, *etc.*)?	☒	☐	The analyses based on simulations match the cryo-ET observations within the discussed scenarios.
**3. Method choice**			
3a. Do simulations contain membranes, membrane proteins, intrinsically disordered proteins, glycans, nucleic acids, polymers, or cryptic ligand binding?	☒	☐	Simulations involve mesoscopic models of membranes and membrane-associated proteins.
3b. Is it described in the text whether the accuracy of the chosen model(s) is sufficient to address the question(s) under investigation (e.g. all-atom vs. coarse-grained models, fixed charge vs. polarizable force fields, implicit vs. explicit solvent or membrane, force field and water model, etc.)?	☒	☐	Mesoscopic models developed for the simulations match the level of details available for the studied system, based on our reported cryo-ET measurements, as well as the data available in the literature, (discussed in the *Material and Methods* section).

3c. Is the timescale of the event(s) under investigation beyond the brute-force MD simulation timescale in this study that enhanced sampling methods are needed?	☐	☒	The timescale of the events (~100 microseconds) are well within the simulation capacity of the presented mesoscopic model.
If **YES**, are the parameters and convergence criteria for the enhanced sampling method clearly stated?	☐	☐	
If **NO,** is the evidence provided in the text?	☒	☐	Suppl. Fig. 8
**4. Code and reproducibility**			
4a. Is a table provided describing the system setup that includes simulation box dimensions, total number of atoms, total number of water molecules, salt concentration, lipid composition (number of molecules and type)?	☐	☒	All the necessary simulation details, considering the solvent-free and mesoscopic nature of the models, are conveniently provided within the text (under *Material and Methods*)
4b. Is it described in the text what simulation and analysis software and which versions are used?	☒	☐	
4c. Are other parameters for the system setup described in the text, such as protonation state, type of structural restraints if applied, nonbonded cutoff, thermostat and barostat, etc.?	☒	☐	
4d. Are initial coordinate and simulation input files and a coordinate file of the final output provided as supplementary files or in a public repository?	☒	☐	Full trajectory files are available from a public repository referenced within the text.
4e. Is there custom code or custom force field parameters?	☒	☐	Response not needed if **No**
If **YES**, are they provided as supplementary files or in a public repository?	☒	☐	Apart from the details included in the *Material and Methods* section, and the publicly available codes, most of the parameters of theses simulations are previously published (cited in the manuscript).

Supplementary references

1 Zhou, Q. *et al.* The primed SNARE-complexin-synaptotagmin complex for neuronal exocytosis. *Nature* **548**, 420-425 (2017). https://doi.org/10.1038/nature23484

2 Colbert, K. N. *et al.* Syntaxin1a variants lacking an N-peptide or bearing the LE mutation bind to Munc18a in a closed conformation. *Proc Natl Acad Sci U S A* **110**, 12637-12642 (2013). https://doi.org/10.1073/pnas.1303753110

3 Stepien, K. P., Xu, J., Zhang, X., Bai, X.-C. & Rizo, J. SNARE assembly enlightened by cryo-EM structures of a synaptobrevin–Munc18-1–syntaxin-1 complex. *Science Advances* **8**, eabo5272 (2022). https://doi.org/doi:10.1126/sciadv.abo5272

4 Grushin, K., Kalyana Sundaram, R. V., Sindelar, C. V. & Rothman, J. E. Munc13 structural transitions and oligomers that may choreograph successive stages in vesicle priming for neurotransmitter release. *Proc Natl Acad Sci U S A* **119** (2022). https://doi.org/10.1073/pnas.2121259119

5 Berndt, A. *et al.* High-efficiency channelrhodopsins for fast neuronal stimulation at low light levels. *Proc Natl Acad Sci U S A* **108**, 7595-7600 (2011). https://doi.org/10.1073/pnas.1017210108

6 Mattis, J. *et al.* Principles for applying optogenetic tools derived from direct comparative analysis of microbial opsins. *Nat Methods* **9**, 159-172 (2011). https://doi.org/10.1038/nmeth.1808

7 Grauel, M. K. *et al.* RIM-binding protein 2 regulates release probability by fine-tuning calcium channel localization at murine hippocampal synapses. *Proceedings of the National Academy of Sciences* **113**, 11615-11620 (2016). https://doi.org/doi:10.1073/pnas.1605256113

8 Chen, T. W. *et al.* Ultrasensitive fluorescent proteins for imaging neuronal activity. *Nature* **499**, 295-300 (2013). https://doi.org/10.1038/nature12354

9 Marvin, J. S. *et al.* An optimized fluorescent probe for visualizing glutamate neurotransmission. *Nat Methods* **10**, 162-170 (2013). https://doi.org/10.1038/nmeth.2333

10 Aggarwal, A. *et al.* Glutamate indicators with improved activation kinetics and localization for imaging synaptic transmission. *Nat Methods* **20**, 925-934 (2023). https://doi.org/10.1038/s41592-023-01863-6

11 Byrd, R. H., Lu, P., Nocedal, J. & Zhu, C. A Limited Memory Algorithm for Bound Constrained Optimization. *SIAM Journal on Scientific Computing* **16**, 1190-1208 (1995). https://doi.org/10.1137/0916069

12 Virtanen, P. *et al.* SciPy 1.0: fundamental algorithms for scientific computing in Python. *Nature Methods* **17**, 261-272 (2020). https://doi.org/10.1038/s41592-019-0686-2

13 Liu, Y. T. *et al.* Isotropic reconstruction for electron tomography with deep learning. *Nat Commun* **13**, 6482 (2022). https://doi.org/10.1038/s41467-022-33957-8

14 Castano-Diez, D., Kudryashev, M., Arheit, M. & Stahlberg, H. Dynamo: a flexible, user-friendly development tool for subtomogram averaging of cryo-EM data in high-performance computing environments. *J Struct Biol* **178**, 139-151 (2012). https://doi.org/10.1016/j.jsb.2011.12.017
