# Supplementary material for: Dynamic nanoscale architecture of synaptic vesicle fusion in mouse hippocampal neurons
Source: Nat Commun. 2025 Dec 13;16:11131. doi: 10.1038/s41467-025-67291-6 (PMC12706093; doi:10.1038/s41467-025-67291-6)
Supplement: Supplementary file 2 — Description of Additional Supplementary File [file 41467_2025_67291_MOESM2_ESM.pdf]

The Description of Additional Supplementary Files

Supplementary Video 1: Tomogram of a stimulated synapse with an open fusion pore.
