# Supplementary material for: Dynamic nanoscale architecture of synaptic vesicle fusion in mouse hippocampal neurons
Source: Nat Commun. 2025 Dec 13;16:11131. doi: 10.1038/s41467-025-67291-6 (PMC12706093; doi:10.1038/s41467-025-67291-6)
Supplement: Supplementary file 4 — Reporting Summary [file 41467_2025_67291_MOESM4_ESM.pdf]

nature portfolio

Corresponding author(s): Jana Kroll, Christian Rosenmund

Last updated by author(s): Oct 17, 2025

Reporting Summary

Nature Portfolio wishes to improve the reproducibility of the work that we publish. This form provides structure for consistency and transparency in reporting. For further information on Nature Portfolio policies, see our Editorial Policies and the Editorial Policy Checklist.

Statistics

For all statistical analyses, confirm that the following items are present in the figure legend, table legend, main text, or Methods section.

n/a	Confirmed	
☐	☒	The exact sample size (*n*) for each experimental group/condition, given as a discrete number and unit of measurement
☐	☒	A statement on whether measurements were taken from distinct samples or whether the same sample was measured repeatedly
☐	☒	The statistical test(s) used AND whether they are one- or two-sided<br>*Only common tests should be described solely by name; describe more complex techniques in the Methods section.*
☒	☐	A description of all covariates tested
☐	☒	A description of any assumptions or corrections, such as tests of normality and adjustment for multiple comparisons
☐	☒	A full description of the statistical parameters including central tendency (e.g. means) or other basic estimates (e.g. regression coefficient) AND variation (e.g. standard deviation) or associated estimates of uncertainty (e.g. confidence intervals)
☐	☒	For null hypothesis testing, the test statistic (e.g. *F*, *t*, *r*) with confidence intervals, effect sizes, degrees of freedom and *P* value noted<br>*Give P values as exact values whenever suitable.*
☐	☒	For Bayesian analysis, information on the choice of priors and Markov chain Monte Carlo settings
☒	☐	For hierarchical and complex designs, identification of the appropriate level for tests and full reporting of outcomes
☐	☒	Estimates of effect sizes (e.g. Cohen's *d*, Pearson's *r*), indicating how they were calculated

Our web collection on statistics for biologists contains articles on many of the points above.

Software and code

Policy information about availability of computer code

Data collection	Cryo-electron tomography tilt series were acquired using SerialEM v4.0 (https://bio3d.colorado.edu/SerialEM/) and PACE-tomo v1.5 (https://github.com/eisfabian/PACEtomo.git). Room temperature and cryo-confocal microscopy data were acquired using LAS X software (Leica microsystems). Clampex 10 (Molecular Devices) was used for electrical field stimulation during electrophysiological recordings and live fluorescence microscopy. For live fluorescence imaging Andor Solis v4.32 (Oxford instruments) was used.
Data analysis	Tomograms were generated using the tomoBEAR pipeline (https://github.com/KudryashevLab/TomoBEAR.git), segmentations were done using MemBrain-seg (https://github.com/teamtomo/membrain-seg.git), Dynamo v1.1 was used for subtomogram generation and averaging (https://www.dynamo-em.org/w/index.php?title=Main_Page), IMOD v4.11 and Fiji v1.54 (https://imagej.net/software/fiji/) were used for morphometric analyses and manual segmentation (https://bio3d.colorado.edu/imod/). All codes developed for analyzing the simulation data, reproducing the corresponding figures and statistics, and constructing the Markov state models are publicly available at: https://github.com/MohsenSadeghi/meso_synaptic_vesicle_fusion. Statistical analyses were performed with Prism v8 (GraphPad) and R v4.5 (https://www.r-project.org/). Graphs were generated using the python packages matplotlib v3.9 (https://matplotlib.org/) and seaborn v0.13 (https://seaborn.pydata.org/) and modified using Affinity Designer v2 (Affinity). 3D visualizations were generated with Visual Molecular Dynamics (https://www.ks.uiuc.edu/Research/vmd/) and ChimeraX v1.8 (https://www.rbvi.ucsf.edu/chimerax/)

For manuscripts utilizing custom algorithms or software that are central to the research but not yet described in published literature, software must be made available to editors and reviewers. We strongly encourage code deposition in a community repository (e.g. GitHub). See the Nature Portfolio guidelines for submitting code & software for further information.

Data

Policy information about availability of data

All manuscripts must include a data availability statement. This statement should provide the following information, where applicable:

- Accession codes, unique identifiers, or web links for publicly available datasets
- A description of any restrictions on data availability
- For clinical datasets or third party data, please ensure that the statement adheres to our policy

All data supporting the findings of this study are available within the paper and its Supplementary Information. The printing scheme for 3D pedestals used in cell culture is provided as source data. Source data referring to graphs and statistical analyses are provided with this paper.

Research involving human participants, their data, or biological material

Policy information about studies with human participants or human data. See also policy information about sex, gender (identity/presentation), and sexual orientation and race, ethnicity and racism.

Reporting on sex and gender	not applicable
Reporting on race, ethnicity, or other socially relevant groupings	not applicable
Population characteristics	not applicable
Recruitment	not applicable
Ethics oversight	not applicable

Note that full information on the approval of the study protocol must also be provided in the manuscript.

Field-specific reporting

Please select the one below that is the best fit for your research. If you are not sure, read the appropriate sections before making your selection.

☒ Life sciences ☐ Behavioural & social sciences ☐ Ecological, evolutionary & environmental sciences

For a reference copy of the document with all sections, see nature.com/documents/nr-reporting-summary-flat.pdf

Life sciences study design

All studies must disclose on these points even when the disclosure is negative.

Sample size	No sample size calculation was performed beforehand. Numbers of independent cell cultures (biological replicates) and of analyzed cells/ synapses per culture (technical replicates) were chosen based on previously reported sample sizes for comparable experimental settings: For imaging of iGluSnFR (live fluorescence microscopy and cryo-confocal microscopy) according to Aggarwal et al. 2023, "Glutamate indicators with improved activation kinetics and localization for imaging synaptic transmission", Nature methods, doi: 10.1038/s41592-023-01863-6; for cryo-ET according to Kusick et al. 2020, "Synaptic vesicles transiently dock to refill release sites", Nature Neuroscience, doi: 10.1038/ s41593-020-00716-1 (Figure 2/whole synapses). We were able to reproduce previous results from electrophysiological recordings, as described in Watanabe et al. 2013, "Ultrafast endocytosis at mouse hippocampal synapses", Nature, doi: 10.1038/nature12809 and therefore repeated the experiment only once.
Data exclusions	For the analysis of fluorescent biosensor kinetics, only acquired datasets with a minimal change in fluorescence intensity of 10% were used for analysis. This was necessary to unambiguously identify the peak fluorescence intensity, on which the analysis of on- and off-kinetics is based. We excluded tomograms from our analysis if the signal-to-noise ratio was too low to unambiguously identify detailed cellular structures. To avoid any bias, this exclusion was done at a low zoom where putative vesicle fusion events were not visible. Additionally, putative vesicle fusion events were excluded in case of doubt after blinding. We added a supplementary figure with examples of excluded putative fusion events and explanations why they were not considered for our quantification.
Replication	We reported all biological and technical replicates in the manuscript. We repeated the combined stimulation and freezing of neurons four times and analyzed changes in fluorescence intensity for each culture separately. All four cultures showed comparable results, confirming the reproducibility of our workflow.
Randomization	During plunge freezings, live imaging and electrophysiological recordings, samples were processed/imaged in blocks, meaning that the different groups/conditions were intermixed. The allocation of samples to groups was done randomly for electrophysiological recordings and live fluorescence microscopoy due to early viral transfection. Allocation to groups for plunge freezings was done based on visual assessment of cell quality to ensure that highest-quality EM grids were used in all groups equally.
Blinding	For blinding during the morphometric analysis of fusion events, these events were preselected by the first author and subtomograms were

Blinding	generated by the second author, whereby the subtomograms were renamed. The renamed subtomograms were used for analysis by the first author. Live imaging experiments at elevated temperature were performed blind. Positions for cryo-confocal microscopy were selected using the fluorophore associated to the channelrhodopsin (YFP or mScarlet) and not the iGluSnFR signal. The analysis of cryo-confocal data was performed blind, meaning maximum intensity projections were generated and randomly intermixed for the selection of positions containing individual neurites. We did not perform blinding during electrophysiological recordings and live imaging at room temperature, because these methods are less prone to bias and the experiments were performed to select the best genetic tools for our study, not to compare phenotypes.

Reporting for specific materials, systems and methods

We require information from authors about some types of materials, experimental systems and methods used in many studies. Here, indicate whether each material, system or method listed is relevant to your study. If you are not sure if a list item applies to your research, read the appropriate section before selecting a response.

Materials & experimental systems

n/a	Involved in the study
☐	☒ Antibodies
☒	☐ Eukaryotic cell lines
☒	☐ Palaeontology and archaeology
☐	☒ Animals and other organisms
☒	☐ Clinical data
☒	☐ Dual use research of concern
☒	☐ Plants

Methods

n/a	Involved in the study
☒	☐ ChIP-seq
☒	☐ Flow cytometry
☒	☐ MRI-based neuroimaging

Antibodies

Antibodies used	Primary antibodies: rabbit anti-Homer1 (Synaptic systems, #160 003, dilution 1:200) and guinea pig anti-Vglut1 (Synaptic systems, #135 304, dilution 1:4,000); secondary antibodies: Rhodamine Red donkey anti-rabbit IgG (Jackson ImmunoResearch, #711-295-152, dilution 1:1000) and Alexa Fluor 647 donkey anti-guinea pig IgG (Jackson ImmunoResearch, #706-605-148, dilution 1:1000)
Validation	rabbit anti-Homer1: specificity for the mouse protein and suitability for immunocytochemistry verified by Synaptic systems; guinea pig anti-Vglut1: specificity shown in K.O., suitability for immunocytochemistry verified by Synaptic systems

Animals and other research organisms

Policy information about studies involving animals; ARRIVE guidelines recommended for reporting animal research, and Sex and Gender in Research

Laboratory animals	0-2 days old C57/BL6/N mice of either sex
Wild animals	No wild animals were part of this study.
Reporting on sex	For neuronal mass cultures, brains from male and female newborn mice were pooled.
Field-collected samples	not applicable
Ethics oversight	All experimental procedures involving the use of mice were approved by the Animal Welfare Committee of the Charité-Universitätsmedizin Berlin and the Berlin State Government.

Note that full information on the approval of the study protocol must also be provided in the manuscript.

Plants

Seed stocks	not applicable
Novel plant genotypes	not applicable
Authentication	not applicable
